# Reply to O. Osibogun’s Letter to the Editor Re: Hess S. et al.; *Nutrients* 2016, *8*, 88

**DOI:** 10.3390/nu8070403

**Published:** 2016-06-29

**Authors:** Sascha Hess, Klaus Eichler

**Affiliations:** Winterthur Institute of Health Economics, Zurich University of Applied Sciences, Winterthur 8401, Switzerland; klaus.eichler@zhaw.ch

In a recent literature review and meta-analysis, we summarized the evidence of reducing anemia in children and adults with fortified condiments and noodles [1]. Here, we respond to a critique of our work published in *Nutrients* (Osibogun O., Letter to the Editor 2016, *8*, 240). We would like to give explanations regarding some of the raised points, as many answers to Osibogun’s comments are provided in our publication.

Osibogun has identified an error in the published Pubmed electronic search strategy (Table 1). We are thankful for the critical reading and apologize for the formatting error in Table 1. The correct Pubmed search strategy reads as follows:

Beside the Pubmed database, we also searched The Cochrane Library, as stated in the Methods section. The additionally mentioned literature database was an Endnote^®^-database of a former project in our university research team about the fortification of milk and cereals.

Osibogun raises concern about the inclusion of fortified noodles. The two included interventions, fortified condiments and noodles, were defined a priori (see inclusion criteria). We included fortified condiments and noodles as commercially distributed fortified foods that fit local nutrition habits may be an additional option to improve nutritional status in developing countries (fortified condiments are widely consumed in Africa and Asia; fortified noodles are specifically consumed in Eastern-Asia). The figure of the study flow summarizes some examples of reasons for exclusion but does not imply that we excluded noodle studies in the course of the review. That we retrieved only one noodle study fulfilling our inclusion criteria highlights the very fact that research needs to be undertaken concerning noodles as a fortified carrier. The evidence of effectiveness for fortified noodles is scarce.

We fully agree with Osibogun that risk of bias assessment and further examination of possible heterogeneity are key methodological elements of sound reviews. Thus, we have performed risk of bias assessment on the study and on the outcome level, pre-specified subgroup analyses, as well as meta-regression analysis to further explain heterogeneity in our review. However, we disagree that examination of publication bias (e.g., via funnel plot or statistical test) is always an inevitable methodological step. The limitations are well known and, due to low statistical power, such testing may indicate no publication bias if only a few primary studies are available, as in our review [2].

Again, we agree with Osibogun that industry funding can lead to bias in reporting results. According to established standards, we have transparently reported our Author Contributions and Author Affiliations (independent university research institute), as well as the role of the funding source (Nestlé Research Centre). Industry funding, however, does not automatically reduce the credibility of our results, which are in line with similar findings of other reviews [3]. Evidence synthesis enables researchers, clinicians and public authorities to make sound decisions. In our opinion, any effective solution is welcome that can contribute to resolve urgent public health problems in the nutrition field.

## Figures and Tables

**Table 1:** Pubmed electronic search strategy. Identified records result from steps 3 and 4.

Step	Pubmed Search
1	Fortif *
2	Condiments (MesH)
	OR Seasoned
	OR Seasoning
	OR Bouillon *
	OR Sprinkle *
	OR Soy sauce *
	OR Fish sauce *
	OR Powder */NOT milk powder *
	OR Noodle *
3	1 AND 2
4	Fortified salt *

Abbrevations: *: Pubmed truncation symbol.
